# Supplementary material for: Hybrid algorithms for generating optimal designs for discriminating multiple nonlinear models under various error distributional assumptions
Source: PLoS One. 2020 Oct 5;15(10):e0239864. doi: 10.1371/journal.pone.0239864 (PMC7535070; doi:10.1371/journal.pone.0239864)
Supplement: Attachment — Submitted filename: comment.pdf [file pone.0239864.s002.pdf]

Subject: Review of Manuscript Number: PONE-D-20-10803
Manuscript Title: Hybrid Algorithms for Generating Optimal Designs for Discriminating Multiple Nonlinear Models under Various Error Distributional Assumptions

Dear Prof.

I wish the best for you.

The article: Hybrid Algorithms for Generating Optimal Designs for Discriminating Multiple Nonlinear Models under Various Error Distributional Assumptions, Included:

Optimal experimental designs require model assumptions to provide the most accurate inference given the available resources. Finding optimal designs that can find the most plausible model among a postulated class is generally a Difficult task because the design criteria require multiple layers of nested optimization.

We propose hybrid algorithms based on particle swarm optimization (PSO) to solve such optimization problems when there are multiple models to discriminate, including cases when the optimal design is singular, the mean response of one or more models are not fully specified and problems that involve four layers of nested optimization.

Using several classical examples, we show the advantages of PSO-based algorithms over current algorithms. As an application, we apply our techniques to discriminate among five nonlinear dose-response models in toxicology. To facilitate use of such designs in practice, a software package is available from the authors to generate various types of discrimination designs and evaluate efficiencies of their competitors.

After carefully reading the article in detail, I came to the conclusion that it was not my field of research, so I cannot review this article accurate completely.
I apologies for this decision.

With the best regard

Mahmoud Afshari, Associate Professor.

Department of Statistics, College of science

Persian Gulf University, 7516913798, Iran

E-mail:afshar.5050@gmail.com or afshar@pgu.ac.ir
