# Supplementary material for: Hybrid algorithms for generating optimal designs for discriminating multiple nonlinear models under various error distributional assumptions
Source: PLoS One. 2020 Oct 5;15(10):e0239864. doi: 10.1371/journal.pone.0239864 (PMC7535070; doi:10.1371/journal.pone.0239864)
Supplement: Attachment — Submitted filename: additional specific responses to 4 reviewers.pdf [file pone.0239864.s006.pdf]

Subject: Review of Manuscript Number: PONE-D-20-10803
Manuscript Title: Hybrid Algorithms for Generating Optimal Designs for Discriminating Multiple Nonlinear Models under Various Error Distributional Assumptions

Dear Prof.

I wish the best for you.

The article: Hybrid Algorithms for Generating Optimal Designs for Discriminating Multiple Nonlinear Models under Various Error Distributional Assumptions, Included:

Optimal experimental designs require model assumptions to provide the most accurate inference given the available resources. Finding optimal designs that can find the most plausible model among a postulated class is generally a Difficult task because the design criteria require multiple layers of nested optimization.

We propose hybrid algorithms based on particle swarm optimization (PSO) to solve such optimization problems when there are multiple models to discriminate, including cases when the optimal design is singular, the mean response of one or more models are not fully specified and problems that involve four layers of nested optimization.

Using several classical examples, we show the advantages of PSO-based algorithms over current algorithms. As an application, we apply our techniques to discriminate among five nonlinear dose-response models in toxicology. To facilitate use of such designs in practice, a software package is available from the authors to generate various types of discrimination designs and evaluate efficiencies of their competitors.

After carefully reading the article in detail, I came to the conclusion that it was not my field of research, so I cannot review this article accurate completely.
I apologies for this decision.

With the best regard

Mahmoud Afshari, Associate Professor.

Department of Statistics, College of science

Persian Gulf University, 7516913798, Iran

E-mail: afshar.SOSO@gmail.com or afshar@pgu.ac.ir

Response: We regret that you were unable to give comments on our work.

General comment

This paper is aimed to propose hybrid-based algorithms for finding optimal discrimination designs with multiple nonlinear models under various error distributional assumptions.
The work is premised on the difficulty of finding optimal discrimination designs that could identify the most plausible model among a postulated class owing to multiple layers of nested optimization as design criteria requirement.

The advantages of the proposed hybrid-based algorithms are demonstrated with examples. The performance of the proposed algorithms is compared with the known algorithms. In addition, the proposed design was applied to discriminate among five nonlinear dose-response models in toxicology. Besides, a R package is developed for generating discrimination optimal designs

The present study addresses an important topic. However, the strength of the manuscript would benefit from attention to the following considerations:

Major comments

The text is littered with grammatical errors and spelling mistakes which make some sentences difficult to understand.
The flow of the reading is made difficult with the use of reference in text as subjects of a sentence, as well as unidentified sections referred to.
The R codes used for generating discrimination optimal designs in this paper is not included.
Statistical theory behind the use of frequencies of success of finding optimal design is not stated.

Responses to comments:
We apologize for the grammatical errors and spelling mistakes. In this revised version, we have tried our best to address these are other issues raised, including all that were identified below. Thank you for taking time to point them out. In the revised manuscript, we have accepted all the comments below and the changes are highlighted in red. We were not aware that this journal does not allow the various sections to be numbered, which in part explains the missing section numbers found in the original submission. In this revision, we have now mostly rephrased the sentence. Following your request, we provide R-codes now but do not address convergence issues of the algorithms. They are very tough theoretical questions that have remained unaddressed satisfactorily by mathematicians to date. What is important is that we work with convex optimization problems and so we can verify the quality of the algorithm-generated designs, including whether they are optimal or not. Our experience with PSO and related algorithms is that they have a high frequency of finding optimal designs or designs with high efficiencies. They are also able to solve many design problems that traditional algorithms cannot find.

Specific comments
Abstract
Recast the objective of the paper in the abstract in line 4-8
Introduction
Line 2-4: not clear! Recast
Line 52: Replace “desigs” with “designs”

Line 53: Replace “devlope” with “develop” Line 54:

Replace “devlope” with “develop”

Line 56-68: The referred sections are not clearly identified in the rest of the text Line 73:

Replace “paraameter” with “parameter”

Maximin T - and KL-optimal Design Criteria

Line 129: What section in particular? As commented above *Hybrid*

Algorithms for Finding Optimal Discrimination Designs Line 148: What

section in particular?

Particle Swarm Optimization

Line 154: PSO to be written in full; do not start a sentence with acronyms Line 154:

Replace “inspipred” with “inspired”

Line 161: Replace “componentwise” with “component-wise” Line

170: Replace “have to have” with “must have”

Line 175: Replace “cpu” with “CPU”

PSO-QN algorithm for Finding an Optimal Design for Discriminating between Two Competing Models

Line 186-187: Replace “an outer one and an inner optimization problem” with “outer and inner optimization problems”

PSO-S-QN algorithm for Finding a Maximin Optimal Design for Discriminating among three or more Models

Firs line of the paragraph should read “This sub-section discusses how we used PSO ideas to find….”

Line 223: Replace “algorthm” with “algorithm”

Line 272: should read “… These findings suggest …”

Line 223: I suggest authors replace “NestedPSO-QN” with “Nested-PSO-QN”, in this line and elsewhere

Standardized Maximin T -optimal Design

Line 318: The statement “… The denominator in the of *T* -efficiency formula…” is unclear Line 319:

What section in particular? As commented earlier and in the rest of the text

Review Report of PONE-20-10803

This work presented Hybrid Algorithms for Generating Optimal Designs for Discriminating Multiple Nonlinear Models under Various Error Distributional Assumptions. The authors used hybrid algorithms optimization approach to tackle multiple models.

All I can say is this article is appropriately fitted within the scope of PLOS ONE. All results are significant and interesting. I highly recommend this paper for publication, although I have few concerns and suggestions given as follows:

1- Important outcomes of the present study must be highlighted in the abstract.

2- In Introduction, author described past work, but little comment on the contribution and shortcoming. Author need to provide critical comments.

3- The considered ranges of governing parameters should be justified. What is the reason for these ranges?

4- Please check the paper again for any possible misprints.

Finally, I think the paper is a great contribution by the authors to the existing literature. We need such serious efforts to improve the quality of the research. The current article with aforesaid changes can be accepted for PLOS ONE. Best of luck.

Responses: Thank you for your generally positive remarks and comments, which we address below.

1. This is now done in the abstract as per your suggestion.
2. We now comment on our contribution and shortcomings in our paper in the abstract and in the Introduction.
3. The governing parameters in the algorithm are mainly the default parameters for the algorithms and other parameters are illustrative. We now make our codes available and so if other parameters are used, the user can easily generate the optimal designs and study sensitivity of the design to parameters or models before implementation.
4. We have now double-checked the entire paper for possible misprints. Thank you for the suggestion.

Many thanks for the encouraging and positive comments of our work.

Title: Hybrid Algorithms for Generating Optimal Designs for Discriminating Multiple Nonlinear Models under Various Error Distributional Assumptions

- Authors investigate different algorithms being used for searching optimal design to discriminate models under various assumptions on error.
- Authors provide a detailed review on T- and KL-optimal Design Criteria and Maximin T- and KL-optimal Design Criteria
- They indicate that run time of the earlier algorithms is not endurable
- They propose hybrid algorithms based on the concept of particle swarm optimization.

Minor Comments

- Page 1 line 3, “apart from the unknown model parameters” should be deleted.
- Page 2 line 3, algorithm should be replaced with algorithms.
- Page 3 line 75, define Θ_i and R^{mi}
- Page 9 line 233, homoscedastic should be replaced with homoscedastic error
- Page 11 line 277, “in that they” should be replaced with “and”.

Final Comments

- Overall idea of the paper is good
- Authors claim to have significantly less run time as compared to earlier algorithms
- The R-code or Mathematica code used to develop algorithms should be provided.

Decision

A minor revision of paper is required in the light of following points:

- Authors should correct typos errors
- Paper may be accepted after minor revision and providing R code to verify results.

Responses: Thank you very much for all your comments. We have carefully incorporated all the above comments in our revised manuscript and changes are highlighted in red. As per your request, we now provide our R-package as supplementary material.
