# Supplementary material for: Hybrid algorithms for generating optimal designs for discriminating multiple nonlinear models under various error distributional assumptions
Source: PLoS One. 2020 Oct 5;15(10):e0239864. doi: 10.1371/journal.pone.0239864 (PMC7535070; doi:10.1371/journal.pone.0239864)
Supplement: Attachment — Submitted filename: Review Report of PONE.docx [file pone.0239864.s004.docx]

**Review Report of PONE-20-10803**

This work presented Hybrid Algorithms for Generating Optimal Designs for Discriminating Multiple Nonlinear Models under Various Error Distributional Assumptions. The authors used hybrid algorithms optimization approach to tackle multiple models.

All I can say is this article is appropriately fitted within the scope of PLOS ONE. All results are significant and interesting. I highly recommend this paper for publication, although I have few concerns and suggestions given as follows:

1- Important outcomes of the present study must be highlighted in the abstract.

2- In Introduction, author described past work, but little comment on the contribution and shortcoming. Author need to provide critical comments.

3- The considered ranges of governing parameters should be justified. What is the reason for these ranges?

4- Please check the paper again for any possible misprints.

Finally, I think the paper is a great contribution by the authors to the
existing literature. We need such serious efforts to improve the quality of the
research. The current article with aforesaid changes can be accepted for PLOS ONE. Best of luck.
