# Supplementary material for: Hybrid algorithms for generating optimal designs for discriminating multiple nonlinear models under various error distributional assumptions
Source: PLoS One. 2020 Oct 5;15(10):e0239864. doi: 10.1371/journal.pone.0239864 (PMC7535070; doi:10.1371/journal.pone.0239864)
Supplement: Attachment — Submitted filename: Final report-PLOS One.docx [file pone.0239864.s005.docx]

Title: Hybrid Algorithms for Generating Optimal Designs for

Discriminating Multiple Nonlinear Models under Various

Error Distributional Assumptions

- Authors investigate different algorithms being used for searching optimal design to discriminate models under various assumptions on error.
- Authors provide a detailed review on T- and KL-optimal Design Criteria and Maximin T- and KL-optimal Design Criteria
- They indicate that run time of the earlier algorithms is not endurable
- They propose hybrid algorithms based on the concept of particle swarm optimization.

**Minor Comments**

- Page 1 line 3, “apart from the unknown model parameters” should be deleted.
- Page 2 line 3, algorithm should be replaced with algorithms.
- Page 3 line 75, define Ө_i_ and R^mi^
- Page 9 line 233, homoscedastic should be replaced with homoscedastic error
- Page 11 line 277, “in that they” should be replaced with “and”.

**Final Comments**

- Overall idea of the paper is good
- Authors claim to have significantly less run time as compared to earlier algorithms
- The R-code or Mathematica code used to develop algorithms should be provided.

**Decision**

A minor revision of paper is required in the light of following points:

- Authors should correct typos errors
- Paper may be accepted after minor revision and providing R code to verify results.
