# Supplementary material for: Hybrid algorithms for generating optimal designs for discriminating multiple nonlinear models under various error distributional assumptions
Source: PLoS One. 2020 Oct 5;15(10):e0239864. doi: 10.1371/journal.pone.0239864 (PMC7535070; doi:10.1371/journal.pone.0239864)
Supplement: Attachment — Submitted filename: PONE-D-20-10803 Reviewer comment.docx [file pone.0239864.s003.docx]

**General comment**

This paper is aimed to propose hybrid-based algorithms for finding optimal discrimination designs with multiple nonlinear models under various error distributional assumptions.

The work is premised on the difficulty of finding optimal discrimination designs that could identify the most plausible model among a postulated class owing to multiple layers of nested optimization as design criteria requirement.

The advantages of the proposed hybrid-based algorithms are demonstrated with examples. The performance of the proposed algorithms is compared with the known algorithms. In addition, the proposed design was applied to discriminate among five nonlinear dose-response models in toxicology. Besides, a R package is developed for generating discrimination optimal designs

The present study addresses an important topic. However, the strength of the manuscript would benefit from attention to the following considerations:

**Major comments**

The text is littered with grammatical errors and spelling mistakes which make some sentences difficult to understand.

The flow of the reading is made difficult with the use of reference in text as subjects of a sentence, as well as unidentified sections referred to.

The R codes used for generating discrimination optimal designs in this paper is not included.

Statistical theory behind the use of frequencies of success of finding optimal design is not stated.

**Specific comments**

*Abstract*

Recast the objective of the paper in the abstract in line 4-8

*Introduction*

Line 2-4: not clear! Recast

Line 52: Replace “desigs” with “designs”

Line 53: Replace “devlope” with “develop”

Line 54: Replace “devlope” with “develop”

Line 56-68: The referred sections are not clearly identified in the rest of the text

Line 73: Replace “paraameter” with “parameter”

*Maximin T - and KL-optimal Design Criteria*

Line 129: What section in particular? As commented above

*Hybrid Algorithms for Finding Optimal Discrimination Designs*

Line 148: What section in particular?

*Particle Swarm Optimization*

Line 154: PSO to be written in full; do not start a sentence with acronyms

Line 154: Replace “inspipred” with “inspired”

Line 161: Replace “componentwise” with “component-wise”

Line 170: Replace “have to have” with “must have”

Line 175: Replace “cpu” with “CPU”

*PSO-QN algorithm for Finding an Optimal Design for Discriminating between Two Competing Models*

Line 186-187: Replace “an outer one and an inner optimization problem” with “outer and inner optimization problems”

*PSO-S-QN algorithm for Finding a Maximin Optimal Design for Discriminating among three or more Models*

Firs line of the paragraph should read “This sub-section discusses how we used PSO ideas to find….”

Line 223: Replace “algorthm” with “algorithm”

Line 272: should read “… These findings suggest …”

Line 223: I suggest authors replace “NestedPSO-QN” with “Nested-PSO-QN”, in this line and elsewhere

*Standardized Maximin T -optimal Design*

Line 318: The statement “… The denominator in the of *T* -efficiency formula…” is unclear

Line 319: What section in particular? As commented earlier and in the rest of the text
